# Exploring IBS in Vietnam: an updated review of current trends and treatment landscape

**DOI:** 10.3389/fmed.2026.1705376

**Published:** 2026-03-16

**Authors:** Thong Duy Vo, Anh Thi Phuong Luu

**Affiliations:** 1 Department of Internal Medicine, School of Medicine, University of Medicine and Pharmacy at Ho Chi Minh City, Ho Chi Minh City, Vietnam; 2 Department of Gastroenterology, University Medical Center Ho Chi Minh City, Ho Chi Minh City, Vietnam; 3 Department of Pharmacology, School of Pharmacy, University of Medicine and Pharmacy at Ho Chi Minh City, Ho Chi Minh City, Vietnam

**Keywords:** irritable bowel syndrome, prevalence, psychological comorbidity, quality of life, Rome IV, scoping review, Vietnam

## Abstract

**Background:**

Irritable bowel syndrome (IBS) is a common functional gastrointestinal disorder with substantial psychosocial and economic impacts. Although research on IBS has expanded in recent years, comprehensive data from low- and middle-income countries, including Vietnam, remain limited. This scoping review provides an updated synthesis of IBS epidemiology, clinical features, diagnostic approaches, treatment strategies, and psychosocial burden in Vietnam.

**Methods:**

Following the Arksey and O’Malley framework and Joanna Briggs Institute guidance, we systematically searched PubMed and Google Scholar for studies published between September 2020 and July 2025 in English or Vietnamese. Eligible studies included original research on IBS prevalence, diagnosis, clinical characteristics, and management in Vietnamese populations. Data were extracted and thematically synthesized across four domains: prevalence/epidemiology, clinical and diagnostic features, management strategies, and psychosocial/economic impacts.

**Results:**

Twenty-one studies met the inclusion criteria, most being cross-sectional hospital-based investigations. Reported prevalence varied widely, ranging from 5.5% among university students to 68.2% in gastroenterology clinic populations. Rome IV criteria were consistently applied in recent studies. IBS-D and IBS-M emerged as the predominant subtypes, with gender and age influencing distribution. Overlaps with *Helicobacter pylori* infection, colonic abnormalities, and small intestinal bacterial overgrowth were frequently documented. Psychological comorbidities, particularly anxiety and depression, were highly prevalent and associated with impaired quality of life. Diagnostic biomarker studies explored fecal calprotectin and anti-vinculin antibodies, although findings remain preliminary. Emerging interventions included probiotics, herbal therapies, sulpiride, and structured counseling programs, all showing improvements in symptoms and quality of life in small-scale trials. However, data on long-term efficacy, dietary interventions, and economic burden remain scarce.

**Conclusion:**

IBS is an increasingly recognized health concern in Vietnam, with high variability in prevalence and strong associations with psychosocial comorbidities. While progress has been made in standardizing diagnostic criteria and initiating interventional studies, critical gaps persist in population-based epidemiology, biomarker validation, pharmacological trials, dietary strategies, and cost-of-illness analyses. Addressing these gaps is essential to optimize IBS care and inform healthcare policy in Vietnam and other resource-limited settings.

## Highlights

- IBS in Vietnam shows highly variable prevalence (5.5–68.2%), with IBS-D and IBS-M being the predominant subtypes, and strong associations with gender, age, and overlapping gastrointestinal conditions (*H. pylori*, SIBO, colonic abnormalities).
- Psychological comorbidities (anxiety, depression) are highly prevalent and significantly impair quality of life, highlighting the need for integrated psychosocial support in IBS management.
- Emerging interventions—including probiotics, herbal therapies, sulpiride, and structured counseling—show promising benefits, but robust population-based studies, cost analyses, and large-scale randomized trials remain critically lacking.

## 1 Introduction

Irritable bowel syndrome (IBS) is a prevalent chronic functional gastrointestinal disorder characterized by a combination of symptoms including abdominal pain, cramping, bloating, gas and changes in bowel habits. It is commonly classified into 4 subtypes based on the predominant bowel pattern: IBS with constipation (IBS-C), IBS with diarrhea (IBS-D), mixed IBS (IBS-M), and unclassified IBS (IBS-U) (1).

Globally, the prevalence of IBS varies widely, ranging from 7% in Southeast Asia and Middle Eastern regions, to 11.8–14.0% in North American, North European and Australia, and to 15.0–21.0% in South Europe, Africa and South America (2). The variation between studies likely reflects differences in diagnostic criteria, study populations, and geographic regions. It may also reflect the influence of potential risk factors-such as genetics, gastrointestinal infections, dietary habits, gut microbiota and psychological comorbidities, which can vary depending on geographical setting.

Despite being non-fatal, IBS places a significant burden on both individuals and society, including direct medical costs as well as indirect social and psychological impacts. In the United Kingdom, approximately £45 million and £25.6 million were spent on selected laxatives and antispasmodics commonly used to treat IBS in primary care (3). A U.S.-based study by Buono et al. reported a substantial economic burden associated with IBS-D, primarily due to increased use of medical services (4). In a separate systematic review, Nellesen et al. estimated that the direct annual cost per patient with IBS ranges from $1,562 to $7,547, while indirect costs, including productivity losses and absenteeism from work, range from $791 to $7,737 per year (5). These findings highlight the dual impact of IBS on both healthcare systems and affected individuals.

Beyond the financial cost, IBS is also associated with a substantial humanistic burden, as it significantly affects patients’ ability to carry out daily activities due to physical discomfort, emotional distress, fatigue, and reduced social engagement. In addition to gastrointestinal-specific anxiety, patients frequently experience depressive symptoms and somatic complaints, all of which are clinically significant and further reduce their quality of life (6).

While the burden of IBS is well-documented in high-income countries, there is a lack of comprehensive data on its prevalence, impact, and management in low- and middle- income settings, including Vietnam. This is concerning given the country’s rapid urbanization, dietary westernization, and increasing rates of stress-related disorders, all of which are known risk factors for IBS (7). Although several small-scale studies have been conducted in Vietnam, they are often limited to specific populations—such as university students or healthcare workers—and differ in both diagnostic criteria and methodology. The most recent scoping review focused on IBS in Vietnam, conducted by Quach et al., summarized prevalence estimates and clinical characteristics across multiple groups. While the study provides a foundational overview of IBS in the country, it does not offer any analysis of direct or indirect economic costs, nor does it address key psychological factors such as anxiety, depression, or quality of life. In addition, the section on management lacks depth, offering only general descriptions of available treatments without evaluating their effectiveness, accessibility or alignment with clinical guidelines (8). Given these gaps, this review aims to (i) provide an updated synthesis of the prevalence, trends, and treatment landscape of IBS in Vietnam based on available literature and (ii) identify key research gaps and propose priorities for future studies and healthcare improvements tailored to the Vietnamese context. Alterations in gut microbiota composition have emerged as a key mechanism underlying irritable bowel syndrome (IBS). These alterations may be driven by multiple factors, including dietary patterns (e.g., low fiber intake, high FODMAP diets), prior antibiotic exposure, post-infectious changes following acute gastroenteritis, psychological stress via the gut–brain axis, and urban lifestyle transitions. Emerging evidence suggests that these factors interact to disrupt microbial diversity, immune regulation, and intestinal barrier function in IBS patients (9, 10). Dysbiosis is associated with increased intestinal permeability, mucosal immune activation, and visceral hypersensitivity, all of which contribute to symptom generation. Probiotics - particularly *Bifidobacterium* and *Lactobacillus* species - play an important role in restoring microbial homeostasis, modulating the gut–brain axis, and improving mucosal barrier function. Several strain-specific probiotics have demonstrated benefits in reducing abdominal pain, bloating, and improving stool consistency. Thus, modulation of gut microbiota represents a promising therapeutic direction for IBS, complementing pharmacologic and psychosocial management strategies.

## 2 Methodology

This review was conducted as a scoping review, following the methodological framework outlined by Arksey and O’Malley and further refined by the Joanna Briggs Institute (JBI). The reporting was guided by the PRISMA Extension for Scoping Reviews (PRISMA-ScR) guidelines.

### 2.1 Rationale for time frame

A recent scoping review by Quach et al. provided a comprehensive summary of studies on IBS in Vietnam published between 1995 and August 2020. To avoid duplication and ensure relevance, this review focus extensively on literature published from September 2020 to July 2025. This period captures recent shifts in diagnostic standards (wider adoption of Rome IV), emerging microbiome-related concepts, and new interventional studies conducted in Vietnam. The goal is to update and extend the current evidence base, identifying new trends, clinical insights, and research gaps.

### 2.2 Objectives

The purpose of this scoping review was to:

- Map the existing literature on the prevalence, clinical characteristics, and treatment landscape, and economic and humanistic impacts of irritable bowel syndrome (IBS) in Vietnam.
- Identify research gaps and areas for future study relevant to Vietnamese healthcare context.

### 2.3 Search strategy

We conducted a comprehensive literature search using PubMed and Google Scholar. Our review is restricted to English or Vietnamese. Search terms included combinations of “*irritable bowel syndrome*,” “*IBS*,” “*Vietnam*,” together with “p*revalence*,” “*epidemiology*,” “*clinical characteristics*,” “*treatment*,” “*quality of life*,” “*economic burden*,” and “*healthcare utilization*,” as well as corresponding Vietnamese terms. Boolean operators (AND/OR) were applied as appropriate.

Additional references were identified through manual screening of reference lists from included studies and relevant reviews.

### 2.4 Eligibility criteria

Inclusion criteria

- Studies conducted in Vietnamese populations
- Publications reporting on IBS prevalence, clinical features, diagnosis, or treatment strategies.
- Peer-reviewed articles with full text available in English or Vietnamese
- Published between September 2020 and July 2025

Exclusion criteria

- Studies unrelated to IBS (e.g., inflammatory bowel disease, colorectal cancer)
- Case reports, editorials review (except to mine references)
- Conference abstracts without full text
- Studies already included in Quach et al.

### 2.5 Study selection

**Figure 1:**
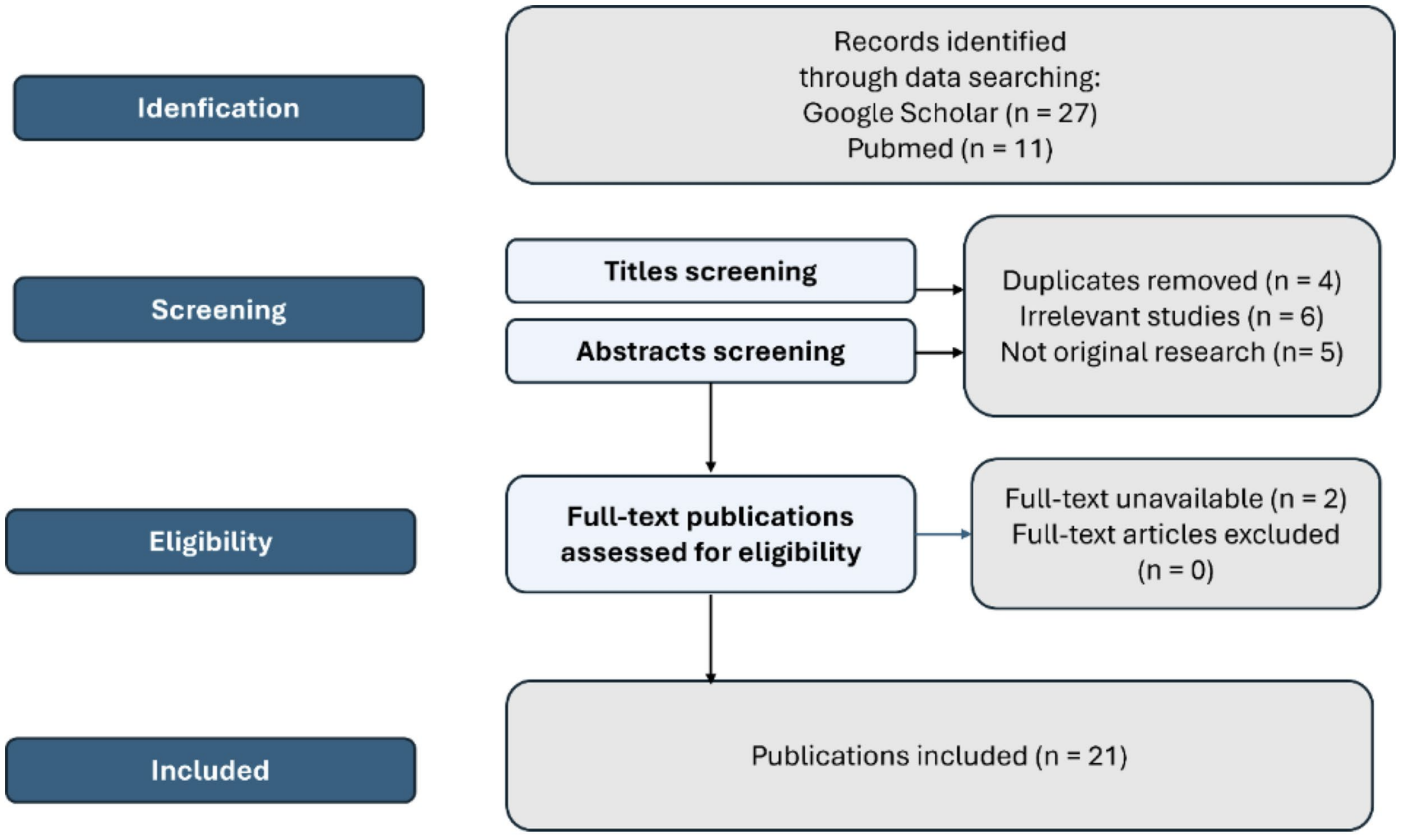
PRISMA flow diagram for the identification and selection of studies.

All records retrieved from the database search were imported into Zotero for reference management, and duplicate entries were removed. Titles and abstracts were screen to assess eligibility based on the predefined inclusion and exclusion criteria. Full-text articles were then retrieved and reviewed in detail. Two reviewers independently screened titles, abstracts, and full texts. Discrepancies were resolved through discussion. The study selection process is summarized in the PRISMA flow diagram (Figure 1).

### 2.6 Data extraction and charting

A standardized data extraction form was used to collect key study information, including:

- Author(s), publication year, study setting
- Population characteristics and sample size
- Diagnostic criteria used (e.g., Rome IV)
- IBS prevalence and subtype distribution
- Treatment approaches and any outcome data
- Study limitations

### 2.7 Data synthesis

Extracted data were summarized descriptively and thematically, and organized into four following domains:

- Prevalence and epidemiological trends
- Clinical characteristics and diagnosis
- Treatment practices and management
- Economic and psychosocial burdens

The findings were compared with those Quach et al. to assess the continuity and changes over time. Gaps in the recent literature were identified to inform future research directions and healthcare planning.

## 3 Results

Of the 21 included studies, 6 were published in English-language international journals, while 15 were published in Vietnamese-language journals. A total of 21 studies, published between September 2020 and July 2025, were included in this review. The majority are cross-sectional in design (*n* = 15), with two randomized controlled trials, two pre-post intervention studies, and three prospective cohort study. Further details regarding the individual studies—including study design, population characteristics, diagnostic criteria, and key findings—are provided in Table 1.

**Table 1:** Summary of included studies assessing IBS in Vietnam.

	Author (Year)	Population, Setting	Study design	Sample size	Diagnostic criteria	Key findings
1	Phan et al. (2025) (14)	IBS outpatients Hue University Hospital	Cross-sectional	186	Rome IV	Mean age 49.5 ± 12.0 years; 53.8% female. IBS-M most common subtype (39.8%), followed by IBS-C (39.2%) and IBS-D (21.0%). Anxiety prevalence: 21.0% (HADS ≥11), 55.9% (HADS ≥8). Depression prevalence: 11.8% (HADS ≥11), 40.8% (HADS ≥8). IBS-C, IBS-D, and IBS-M linked with higher depression risk (OR 4.26–7.01, *p* < 0.001). Men less likely to have depression than women.
2	Thai et al. (2025) (32)	IBS outpatients with poor QoL score Soc Trang Provincial General Hospital	Randomized controlled trial	77 (monthly counseling) 81 (control)	Rome IV	After 3 months of monthly counseling, QoL improved in 32.5% of intervention vs. 7.4% of control group; intervention effectiveness: +25.1% (*p* < 0.001).
3	Ho et al. (2024) (15)	IBS patients with *H. pylori–positive* gastric/duodenal ulcers Bac Lieu General Hospita and Bac Lieu Military Hospital	Cross-sectional	77	Rome IV	Average age 45.6 ± 15.1, men accounted for a higher proportion than women. 100% of patients had symptoms of abdominal pain at least once per week in the last 3 months. Abdominal pain along the colonic framework accounts for 57.1%. Changes in stool characteristics accounted for 90.9% IBS-D accounted for a high proportion (46.8%). Colonoscopy detected lesions in 14.3%.
4	Dinh et al. (2024) (17)	IBS patients 175 Military Hospital, Ho Chi Minh City	Cross-sectional	163	Not mention	Mean age 55.7 ± 15.7 years; 58.3% male; age ≥ 60 most common (44.2%). Anxiety disorders significantly more common in lower education groups. Risk factors: low education, unclear disease understanding, poor treatment adherence, specific fear. Weak positive correlation between Hamilton score and duration of anxiety symptoms.
5	Bui et al. (2024) (23)	Adult IBS-D patients, Three tertiary hospitals in Hanoi	Cross-sectional	215 (IBS-D) 15 (control) 71 (UC) 18 (CD)	Rome IV	Median fecal calprotectin: healthy 20.0 μg/g, IBS-D 17.7 μg/g, UC 1710.0 μg/g, CD 560.5 μg/g. Median CRP: IBS-D 1.3 mg/L, UC 7.0 mg/L, CD 10.1 mg/L. Median IL-6: IBS-D 2.3 pg./mL, UC 16.8 pg./mL, CD 9.4 pg./mL. Biomarkers significantly higher in IBD vs. IBS-D (*p* < 0.05). Cut-offs for differentiating IBD vs. IBS-D: + Calprotectin 110.5 μg/g (Sn 93.3%, Sp 91.4%) + IL-6 7.2 pg./mL (Sn 92.0%, Sp 78.0%) + CRP 2.4 mg/L (Sn 83.3%, Sp 86.0%)
6	Nguyen and Nguyen (2024) (24)	Patients with IBS and IBD Thai Nguyen National Hospital	Cross-sectional	24 (IBD) 28 (IBS)	Rome IV	Median fecal calprotectin: IBS 25.7 μg/g, IBD 87.0 μg/g. Higher levels associated with fever, bloody stools, and UC activity (Mayo score). No association with weight loss, CD activity (CDAI), or IBS subtype. Cut-off 52.25 μg/g differentiated IBD from IBS (Sn 87.5%, Sp 82.1%, AUC 0.92).
7	Truong et al. (2024) (25)	Adult IBS patients Medic Clinic, Ho Chi Minh City	Retrospective cross-sectional	300	Rome IV	Positive FOBT (fecal occult blood test) and calprotectin ≥ 50 μg/g indicate a warning risk of developing organic disease 2–8 times higher (*p* < 0.05) in IBS patients. FOBT had higher sensitivity while fecal calprotectin had higher specificity.
8	Nguyen et al. (2024) (26)	IBS-D patients vs. non-IBS group Thanh Nhan Hospital, Hanoi	Cross-sectional	251 (IBS), 31 (control)	Rome IV	Anti-vinculin level is significantly higher in the IBS group (*p* < 0.05).
9	Tran et al. (2023) (11)	Medical students Ho Chi Minh City	Cross-sectional	400	Rome IV	400 medical students; FGIDs prevalence 10.3% (FD 6.5%, IBS 5.5%); overlap syndrome 3.0%. GAD prevalence 6.8%, MDD prevalence 10.2%. Urinary test positive in 45%. MDD strongly associated with FGIDs (OR 5.60, 95%CI 2.17–14.43, *p* < 0.001) and overlap syndrome (OR 10.08, 95%CI 2.24–45.27, *p* = 0.003).
10	Ho et al. (2023) (19)	IBS-D patients GI Research Institute, Hanoi	Cross-sectional	30	Rome IV	Prevalence of SIBO in IBS-D patients: 70%. Baseline and early hydrogen concentrations (15–45 min) significantly higher in SIBO vs. non-SIBO (*p* < 0.05). No differences in age, sex, BMI, stool frequency, or IBS severity between groups. SIBO group had more abdominal distension and poorer quality of life (*p* < 0.05)
11	Pham et al. (2023) (12)	IBS patients Ca Mau General Hospital, Ca Mau	Prospective cross-sectional	110	Rome IV	Prevalence of IBS in gastroenterology clinic (Ca Mau General Hospital): 68.2%. Subtypes: IBS-D 29.3%, IBS-C 28.0%, IBS-M 13.4%, IBS-U remainder. 57.3% female. After treatment, proportion with moderate–good QoL increased from 6.7 to 98.7%.
12	Phan et al. (2023) (13)	IBS patients Hue University of Medicine and Pharmacy Hospital, Hue	Cross-sectional	287	Rome IV	Mean age 50.9 years; 51.0% female. IBS subtypes: IBS-C 25.4%, IBS-D 13.6%, IBS-M 25.8%, IBS-U 35.2%. HADS: anxiety disorders (AD) 43.6%, depressive disorders (DD) 30.3%. Females more likely to have AD (OR 1.66) and DD (OR 1.96) than males (*p* < 0.05). Compared to IBS-U: AD more common in IBS-C (OR 4.37), IBS-D (OR 4.44), IBS-M (OR 5.59); DD more common in IBS-C (OR 4.26), IBS-D (OR 7.01), IBS-M (OR 6.59) (all *p* < 0.05).
13	Keo et al. (2022) (16)	Patients with undergoing colonoscopy Can Tho University Hospital, Can Tho	Cross-sectional	187	Rome IV	Average age group 50–59 years; 53.5% female. IBS subtypes: IBS-D 49.7%, IBS-C 44.4%, IBS-M 4.8%, IBS-U 1.1%. 187 patients underwent colonoscopy; 54% had lesions. + Of these: 60.3% with alarm features, 41.0% without alarm features. + Lesion types: colitis 17.6%, polyps 36.9%, diverticula 13.4%.
14	Trinh et al. (2023) (29)	IBS-D patients	Pre-post intervention	45	Rome IV	45 IBS patients. Clinical symptoms, disease severity, and QoL improved over treatment period. Use of rigid capsule “Central supplement” reduced clinical symptoms and improved quality of life.
15	Truong et al. (2024) (25)	Adult IBS patients (Rome IV) Medic Clinic, Ho Chi Minh City	Retrospective cross-sectional	300	Rome IV	Positive fecal occult blood test (FOBT) and fecal calprotectin ≥ 50 μg/g were associated with a 2–8-fold increased risk of organic gastrointestinal disease (*p* < 0.05). FOBT showed higher sensitivity, while fecal calprotectin demonstrated higher specificity in identifying patients requiring further investigation.
16	Nguyen et al. (2022) (18)	IBS patients Bach Mai Hospital, Hanoi	Prospective, cross-sectional	207	Rome IV	Overall QoL score: 79.3 (95% CI: 77.2–81.3). Lowest domains: dietary restriction 62.3, physical activity 67.9. Highest domains: social activities 90.6, social relationships 94.0. QoL distribution: very poor 5.8%, poor 20.3%, moderate 42.0%, good 31.9%. Sexual activity QoL: highest in patients >70 years (100), lowest in <30 years (77.8).
17	Tran et al. (2022) (33)	Patients underwent colonoscopy with IBS-like symptoms Nguyen Tri Phuong Hospital, Ho Chi Minh City	Cross-sectional	265	Rome IV	265 cases underwent colonoscopy; 163 (61.5%) had ROME IV IBS-like symptoms. Among IBS-like cases: 41.7% normal colonoscopy; 95 with lesions (colitis 33.7%, adenoma 9.8%, colorectal cancer 3.7%). In patients without alarm features: low rate of anatomic abnormalities; no advanced neoplasms detected. Predictive models for colorectal advanced neoplasms: + Age (OR 1.07), rectal bleeding (OR 10.47), weight loss (OR 7.74). + Rectal bleeding (OR 7.47), weight loss (OR 1.41), APCS score (OR 2.00).
18	Ermolenko et al. (2022) (30)	IBS patients Hanoi	Pilot interventional study	11	Not mention	Probiotic L3 treatment improved clinical symptoms in IBS patients. Gut microbiota changes: ↑ *Firmicutes* (*Roseburia, Blautia*); ↓ *Enterobacteriaceae*. α-diversity decreased after treatment. Microbiota changes consistent with prior findings in Russian populations.
19	Vo and Nguyen (2021) (27)	IBS outpatients University Medical Center Ho Chi Minh City	Prospective cohort study	246	Rome III	Sulpiride users showed improved QoL over 8 weeks compared to non-users (*p* < 0.001)
20	Vu et al. (2021) (28)	IBS-D patients Traditional Medicine Clinic, Hanoi	Pre-post intervention	45	Rome IV	After treatment: 88.6% good/quite effective outcomes. Clinical improvement in abdominal pain, loose stools, bloating, stool disorder. 92.9% reported no QoL impairment. 82.2% had no or only mild colonic dysfunction. HCR1 capsules significantly improved symptoms and traditional medicine conditions (*p* < 0.05).
21	Dao et al. (2021) (20)	Patients with chronic GI symptoms, anxiety and depression Hoang Long Clinic, Hanoi	Single-center uncontrolled trial	83	Rome IV	9.6% of patients with depression/anxiety diagnosed with IBS. HADS scores improved over time: baseline 20.0 (6.3) → 1 month 7.2 (5.4) → 2 months 4.9 (5.1). QoL improved significantly after probiotic (8 bacterial strains). Adverse events: <5% mild symptoms (fullness, diarrhea, sleep complaints).

Most studies were conducted in hospital-based setting within major urban centers, including Ho Chi Minh City, Hanoi, Hue, and Can Tho. Study populations consisted primarily of IBS outpatients, and university students, and individuals assessed for other gastrointestinal conditions, including *Helicobacter pylori*-associated ulcers and inflammatory bowel disease (IBD).

Diagnostic approaches were relatively consistent across studies, with 17 out of 19 studies employing Rome IV criteria. Sample sizes ranged from 11 to over 400 participants.

Thematically, the studies primarily focus on four key areas:

(1) IBS prevalence, subtype distribution and clinical characteristics.
(2) Psychological comorbidities and their association with quality of life.
(3) Diagnostic biomarkers.
(4) Treatment strategies and intervention outcomes.

### 3.1 Prevalence and subtype distributions of IBS in Vietnam

The reported prevalence of irritable bowel syndrome (IBS) in Vietnam varies considerably depending on the study population and setting. A relatively low prevalence of 5.5% was reported in a study conducted among newly enrolled medical students in Ho Chi Minh City (11). In contrast, significantly higher rates were observed in clinical populations. For instance, a prevalence of 68.2% was recorded at Ca Mau General Hospital (12), while another study reported a prevalence of 14.4% at Hue University of Medicine and Pharmacy Hospital (13). Notably, a more recent study by Phan et al., conducted at the same hospital, documented a higher prevalence of 35.5%, suggesting either an increasing trend or sampling variation across patient groups (14).

Across studies reporting IBS subtype distribution, IBS-D and IBS-M were the most commonly observed forms. IBS-D was identified as the most prevalent subtype in three studies, with the highest proportion reported to be 49.7% (12, 15, 16). Two studies found IBS-M to be the most common subtype in two studies by Phan et al. at Hue University of Medicine and Pharmacy Hospital, with reported rates ranging from 21 to 32.6% (13, 14). The prevalence of IBS-C varied from 25.4 to 44.4% across settings (13–16), while IBS-U was consistently the least common (12, 13, 16), with proportions between 1.1 and 35.2%. Rome IV was consistently used in all studies reporting prevalence and subtype distributions, enhancing the comparability of results across settings.

Several studies reported differences in IBS prevalence according to gender. Four studies found a higher prevalence of IBS in females, with rates ranging from 51 to 57.3% (12–14, 16). In contrast, studies by Dinh et al., which focused on IBS patients with *Helicobacter pylori* infection, and Ho et al. observed higher rates in males (15, 17). The association between gender and IBS subtypes in Vietnam was only reported in one study, which found that IBS-M was the most common among females (39.8%), followed by IBS-C at 39.2%. On the other hand, males were more likely to have IBS-D (14).

IBS prevalence tends to be higher in older adults. In a study by Dinh et al., 44.2% of the studied population was more than 60 years old (17). Additionally, other studies also recorded the average age of IBS onset to be typically around 50 years old (11, 14). Ho et al. and Nguyen et al. reported a slightly lower average age, at 45 and 47 years old, respectively (15, 18). In younger populations, such as university students, the prevalence is generally lower (11), with a rate of 5.5%.

### 3.2 Gastrointestinal overlapping conditions in IBS patients in Vietnam

Several studies included in this review have reported an overlap between IBS and other gastrointestinal conditions, particularly *Helicobacter pylori* infection, structural abnormalities detected through colonoscopy, and small intestinal bacterial overgrowth (SIBO).

In a study by Ho et al., patients with *H. pylori*-associated gastric or duodenal ulcers commonly exhibited the IBS-D subtype. Within this group, 57.1% reported colonic pain, 90.9% had altered stool consistency, and 14.3% presented with colonoscopic abnormalities (15). Similarly, Keo et al. detected organic findings in 54% of patients undergoing colonoscopy for presumed IBS symptoms—despite the absence of alarm features. Identified abnormalities include polyps (36.9%), inflammation or ulcers (17.6%), and diverticula (13.4%) (16). SIBO has also emerged as a relevant comorbidity. In Ho et al., the prevalence of SIBO among IBS-D was 70%. These patients reported more severe bloating and significant lower quality of life compared to those without SIBO (19).

### 3.3 Psychological comorbidities and quality of life of IBS patients in Vietnam

Several studies have shown a significant association between IBS and psychological factors such as anxiety and depression. In 2021, Dao et al. reported that 9.6% of IBS patients were diagnosed with both depression and anxiety (20). Risk factors for these conditions include gender, IBS subtypes, and education level. Two studies found that women with IBS were more likely to experience anxiety and depression compared to men. Anxiety disorders were also more prevalent in patients with IBS-C, IBS-D, and IBS-M compared to those without IBS (13, 14). Furthermore, Dinh et al. found that anxiety was more common among IBS patients with a lower education level and those who had an unclear understanding of the disease (17).

Quality of life (QOL) is significantly lower in individuals diagnosed with IBS. A cross-sectional study by Ho et al. found that 70% of patients with IBS-D are diagnosed with SIBO, which is highly associated poorer QOL (15). Similarly, 42% of the patients in a study by Nguyen et al. had moderate QOL. In terms of specific domains, the area of dietary restriction had the lowest QOL score, followed by the physical activity domain. The QOL score on sexual activity in the group of 70 years old and above is higher than other age groups, the lowest is in the age group under 30 years old (18).

### 3.4 Diagnostic tools for IBS in Vietnam

#### 3.4.1 Rome IV diagnostic criteria

Since 2020, Rome IV criteria has been consistently used across studies reviewed, providing a standardized approach for diagnosing IBS. This is important because it ensures the comparability of results between different studies. The Rome IV criteria state that for a diagnosis of IBS, recurrent abdominal pain must occur at least 1 day per week in the last 3 months, and is associated with defecation changes (frequency, consistency). These criteria must be fulfilled for the last 3 months, with symptom onset at least 6 months prior to diagnosis, which help differentiate IBS from other gastrointestinal disorders (21).

#### 3.4.2 Diagnostic biomarkers

Fecal calprotectin has been explored as a potential biomarker to distinguish IBS from inflammatory bowel disease (IBD) (22). Bui et al. reported significantly higher levels of fecal calprotectin in IBD patients (including those with ulcerative colitis and Crohn’s disease) compared to patients diagnosed with IBS-D (23). Conversely, Nguyen et al. found no significant relationship between calprotectin levels and IBS subtypes, suggesting limited utility of this biomarker in IBS diagnosis (24).

Truong et al. indicated that a positive FOBT (fecal occult blood test) and calprotectin levels ≥ 50 μg/g serve as a warning sign, indicating a 2–8 times higher risk (*p* < 0.05) of developing an organic disease in IBS patients, suggesting the need for further investigation when these markers are present (25).

The optimal cut-off points of fecal calprotectin to differentiate IBS from IBD was reported in two studies. In Bui et al., the cut-off was 110.5 μg/g, with sensitivity and specificity of 93.3 and 91.4%, respectively (23). In contrast, Nguyen et al. reported a lower cut-off of 52.25 μg/g, with sensitivity and specificity of 87.5 and 82.1%, respectively (24).

Another emerging biomarker is anti-vinculin, which was reported in a study by Nguyen et al. The study found that anti-vinculin levels were significantly higher in IBS patients, suggesting its potential role as a marker for IBS diagnosis (26).

### 3.5 Interventions for IBS in Vietnam

#### 3.5.1 Pharmacological treatments

Three studies have investigated the efficacy of emerging pharmacological treatments for IBS, including sulpiride, a D_2_-receptor-blocking antipsychotic medication, an herbal solid pill “Central Supplement,” and an herbal capsule HCR1. A prospective cohort study by Vo and Nguyen compared patients prescribed with sulpiride with non-users, reporting significant improvement in patients’ quality of life over 8 weeks. The study demonstrated that sulpiride had positive effects on multiple aspects of patients’ life, especially in domains such as comfortable characteristics, impeded performance, physical fitness, health anxiety, social reactions and relationships (27). In the same year, Vu et al. conducted a pre-post study evaluating effectiveness of an herbal capsule HCR1 on 70 IBS patients and symptoms were evaluated immediately after surgery, at the time of discharge, and 3 months after surgery. Clinical symptoms such as abdominal pain and quality of life were reported improved in 82.2 and 92.9%, respectively (28). Another prospective pre-post study by Trinh et al. evaluated the effectiveness of a rigid capsule “Central Supplement,” which contains herbal ingredients, in patients with IBS-D. The results indicated that the clinical symptoms, disease severity, and quality of life of all patients included in the study improved considerably over the course of treatment (29).

#### 3.5.2 Probiotics and gut microbiota

Two studies have evaluated the efficacy of probiotics in IBS patients. One pilot interventional study conducted by Ermolenko et al. involving 11 IBS patients using probiotic L3 reported significant improvement in clinical symptoms, along with a shift in gut microbiota. The study found an increase of *Firmicutes* (particularly *Roseburia*, *Blautia*) and a decrease in *Enterobacteriaceae* (30). Another study by Dao et al., focusing on IBS patients with concomitant anxiety and depression, found that their quality of life also improved significantly after using a probiotic product containing eight bacterial strains. The probiotics were generally well-tolerated, with only under 5% of patients developed mild adverse effects such as fullness, diarrhea or sleep disturbances (20). These findings are in line with recent global meta-analyses demonstrating strain-specific benefits of probiotics in IBS.

#### 3.5.3 Counseling and psychological interventions

Two studies have assessed the efficacy of counseling interventions for IBS patients, particularly those with poor quality of life (QoL). In 2021, Vo et al. conducted a randomized controlled trial with 8-week follow-up involving 273 IBS patients at the University of Medicine and Pharmacy Hospital in Ho Chi Minh City. Of these, 132 patients received educational sessions with a clinical pharmacist, which covered IBS knowledge, lifestyle changes, diet, and medication adherence. Patients were also provided with educational materials and received telephone consultations for reinforcing information and offering further advice on lifestyle modifications and adherence to medication. The study reported significant improvements in QoL in the intervention group compared to the non-intervention group, particularly in areas such as discomfort, hindrance of activities, physical health, health anxiety, social reactions, and relationships. A positive relationship between counseling and improvements in QoL was confirmed (31).

In 2025, Thai et al. conducted another randomized controlled trial in IBS patients at Soc Trang General Hospital. In this study, patients received monthly counseling from doctors and nurses, which included disease education, dietary advice, and exercise recommendations. After 3 months, the intervention group showed significant improvement in QoL, with a 25.1% improvement compared to the control group. This study further supports the effectiveness of routine counseling in enhancing the quality of life for IBS patients (32).

Among the emerging interventions for IBS, probiotics have gained increasing attention in both Vietnamese and international research due to their potential to modulate gut microbiota and improve gastrointestinal symptoms. Table 2 summarizes available probiotic studies conducted in Vietnam alongside representative global evidence, highlighting strain-specific characteristics, proposed mechanisms, and clinical outcomes. These findings support the concept that targeted probiotic therapy may offer complementary benefits in symptom control, microbiota restoration, and quality-of-life improvement for IBS patients.

**Table 2:** Summary of probiotic interventions for IBS in Vietnam and related global evidence.

Study	Population/design	Probiotic strain(s)	Mechanism/rationale	Outcomes	Adverse effects
Dao et al., 2021 (*J Multidiscip Healthc*) (20)	IBS with anxiety/depression, Vietnam	Mix of 8 strains (*Lactobacillus acidophilus*, *L. plantarum*, *Bifidobacterium bifidum*, etc.)	Restores gut–brain axis balance, reduces inflammation	Improved QoL and anxiety/depression scores	Mild GI discomfort (<5%)
Ermolenko et al., 2022 (*Exp Clin Gastroenterol*) (30)	11 IBS-D patients, Vietnam	*Enterococcus faecium* L3	↑ *Roseburia*, *Blautia*; ↓ *Enterobacteriaceae*	Symptom relief and microbiota normalization	None significant
Yang R et al., 2024 (*Clin Nutr ESPEN*) (9)	Global IBS population – Systematic review & meta-analysis	Multiple species (*Lactobacillus, Bifidobacterium, Saccharomyces boulardii)*	SCFA production, immune modulation, barrier enhancement	Significant symptom reduction and improved stool regularity	Well tolerated across studies
Dicks LMT, 2022 (*Microorganisms*) (10)	Review on neuro-microbiota interaction	*Bifidobacterium* and *Lactobacillus* species	Gut–brain neurotransmitter regulation (GABA, serotonin), anti-inflammatory signaling	Supports mechanistic link between probiotics and IBS symptom relief	-

Adapted from Vietnamese and international data for comprehensive comparison.

**Figure 2:**
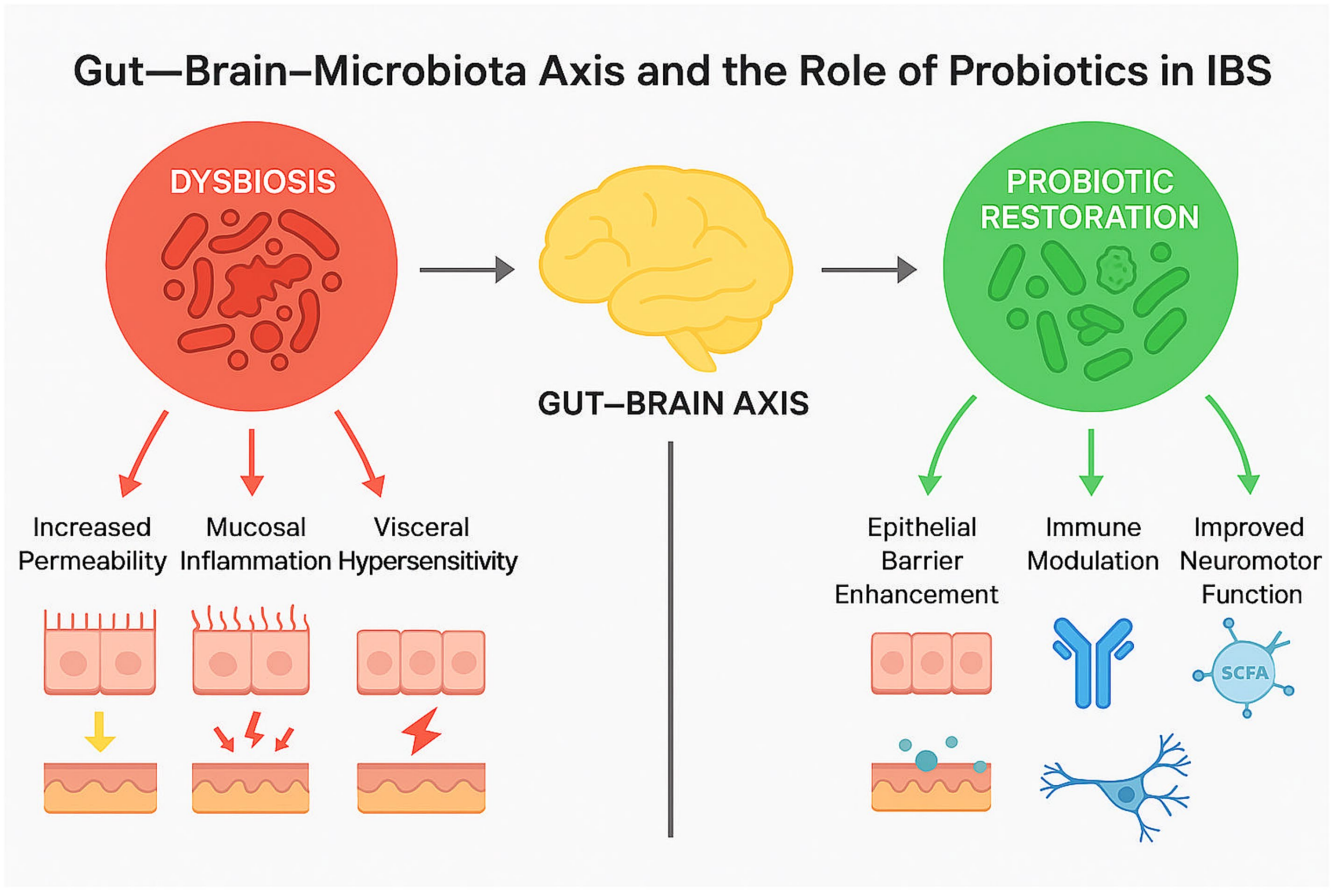
Gut–brain–microbiota axis and the role of probiotics in IBS. The figure depicts how dysbiosis leads to increased permeability, mucosal inflammation, and visceral hypersensitivity; probiotics restore balance through epithelial barrier enhancement, immune modulation, SCFA production, and improved neuromotor function.

The dynamic interaction between intestinal dysbiosis, the gut–brain axis, and probiotic modulation is summarized in Figure 2. This schematic highlights how microbiota imbalance contributes to intestinal permeability, mucosal inflammation, and visceral hypersensitivity, while probiotics restore balance through multiple mechanisms-enhancing epithelial barrier integrity, regulating immune responses, producing short-chain fatty acids (SCFAs), and improving gut–brain communication. Together, these mechanisms underline the multifaceted role of probiotics in the pathophysiology and management of IBS.

## 4 Discussion

This updated scoping review builds upon the foundational work of Quach et al. (8), which summarized key insights into the prevalence, clinical characteristics, and management of irritable bowel syndrome (IBS) in Vietnam based on studies published between 1995 and 2020. Despite providing a valuable starting point, that earlier review identified substantial limitations in the existing literature, including the absence of national prevalence data, inconsistency in diagnostic criteria, and a lack of interventional research.

### 4.1 Probiotic modulation of gut microbiota: strain-specific insights and global perspectives

Recent studies have highlighted the pivotal role of gut microbiota modulation in IBS pathophysiology. Both Vietnamese and international research show that probiotic efficacy is strain-dependent. *Lactobacillus plantarum* 299v and *Bifidobacterium infantis* 35,624 have been reported to improve abdominal pain and stool consistency through modulation of inflammatory cytokines and mucosal integrity. Local Vietnamese trials using mixed or enterococcal probiotics demonstrated beneficial microbial shifts, notably increases in Roseburia and Blautia species—genera associated with short-chain fatty acid production and mucosal health.

These findings align with global meta-analyses suggesting that probiotics may act through several mechanisms, including immune regulation, production of short-chain fatty acids, modulation of the gut–brain axis, and restoration of barrier function. However, strain specificity remains critical, and future trials in Vietnam should adopt well-characterized probiotic strains and standardized outcome measures to better define their therapeutic potential.

Between September 2020 and July 2025, there has been a marked expansion in both the quantity and quality of IBS research in Vietnam. Notably, several of the methodological and conceptual limitations noted in the previous review have been directly addressed in newer studies.

First, there has been a clear trend toward standardized diagnostic practices, particularly the adoption of the Rome IV criteria. Of the 19 studies included in this review, 17 utilized Rome IV, with the remaining two (published in 2020 and early 2021) still applying Rome III. This represents a significant departure from the earlier literature, in which Rome I–III and even Manning criteria were applied inconsistently. The shift toward Rome IV enhances the internal validity and cross-study comparability of findings, particularly in relation to IBS subtype classification.

Second, the scope of prevalence studies has broadened. While Quach et al. reported IBS prevalence rates of 7.2 to 14.4% based on select populations (e.g., university students, healthcare workers), recent research has identified a wider range—from 5.5% among student cohorts to 68.2% in hospital-based populations. The latter also correlates with a shift in age distribution, with the average patient age now reported between 45 and 50 years—higher than the previously documented range of 27–38 years. Regarding subtype patterns, although IBS-D remains predominant, there is growing recognition of IBS-M, particularly among female patients. Some newer studies have also analyzed subtype distributions by sex, revealing a higher prevalence of IBS-M and IBS-C among women, whereas IBS-D appears more common in men - an analytical depth not present in the 2021 review by Quach et al. The wide prevalence range observed in Vietnam is comparable to global variations reported across Asia and Western countries, largely driven by differences in diagnostic criteria, study settings, and health-seeking behavior.

Third, this review highlights a substantial overlap between IBS and other gastrointestinal conditions, particularly *H. pylori* infection, structural abnormalities, and SIBO, which were not comprehensively addressed in the earlier study. Recent studies have documented a high prevalence of IBS-D subtype in patients with *H. pylori*-related ulcers, with a high incidence of colonic abnormalities in IBS patients undergoing colonoscopy, even without alarm features. In contrast, the 2021 review by Quach et al. only briefly mentioned the need for careful differentiation between IBS and other conditions like IBD and colorectal cancer, but did not explore the specific overlaps with *H. pylori* and colonoscopy findings in depth. Additionally, SIBO emerged as a clinically relevant comorbidity, with one study reporting a prevalence of of SIBO in IBS-D patients, who also demonstrated a lower quality of life. While Quach et al. acknowledged the existence of SIBO, its epidemiological and clinical significance in the Vietnamese context was not emphasized. The predominance of IBS-D and IBS-M observed in Vietnamese cohorts is consistent with reports from other Asian populations, while sex-related differences resemble patterns reported in Western studies.

Fourth, the psychosocial impacts of IBS are increasingly emphasized. While the earlier review included only limited data linking IBS with anxiety, depression, or quality of life (QoL), the current literature reflects a more comprehensive approach. Multiple studies have utilized validated instruments to measure QoL and psychological comorbidities, revealing strong associations between IBS and stress-related factors, especially in women, individuals with lower educational attainment, and those with limited understanding of the disease. The strong association between IBS, anxiety, depression, and impaired quality of life observed in Vietnam mirrors findings from Europe and North America, supporting the biopsychosocial model of IBS.

Fifth, advancements in biomarker research are notable. Quach et al. only briefly mentioned CRP and calprotectin as potential differential tools to rule out inflammatory bowel disease (IBD). However, more recent studies have identified calprotectin cut-off thresholds (e.g., 110.5 μg/g) with high sensitivity and specificity. Additionally, anti-vinculin antibodies have emerged as a novel biomarker in at least one Vietnamese cohort study, although findings remain preliminary.

Lastly, there has been a transition from descriptive research to interventional trials. The 2021 review noted the theoretical use of pharmacologic agents - such as antispasmodics and probiotics - but no efficacy data from Vietnamese populations. In contrast, the current review incorporates several controlled trials and prospective cohorts evaluating interventions ranging from sulpiride and herbal remedies to probiotics and pharmacist-led counseling programs. Notably, structured patient education and counseling - whether provided by pharmacists or physicians - were consistently associated with improvements in QoL, suggesting a promising approach for integrating psychosocial support into routine IBS care.

## 5 Future directions

Despite the progress made in understanding the prevalence, clinical characteristics, diagnosis, and treatment of IBS in Vietnam, there are still significant gaps in knowledge and treatment practices. The following areas should be prioritized for future research and healthcare improvement:

### 5.1 Population-based studies

The current studies on IBS in Vietnam are often limited to specific populations (e.g., medical students, hospital patients) and regions. Community-based research should be conducted to gain better understanding of prevalence of IBS across the general population. Future studies should also focus on broader age groups and more diverse populations, considering the observed gender differences and age-related trends in IBS prevalence.

### 5.2 Biomarkers

There is a need to further investigate potential biomarkers in IBS, such as calprotectin and anti-vinculin to differentiate IBS from other structural gastrointestinal conditions, particularly in clinical settings.

### 5.3 Pharmacological interventions

Robust evidence regarding the pharmacological management of IBS in Vietnam are limited, with most available studies being cohort studies or pilot trials. Preliminary data have suggested the efficacy of treatments like sulpiride and herbal supplements. However, these findings are constrained by the lack of long-term efficacy and safety data. Therefore, there is a critical need for well-designed randomized controlled trials in Vietnam to evaluate both efficacy and safety of commonly used medications in the Vietnamese context. This includes pharmacological agents such as antispasmodics, probiotics, and tricyclic antidepressants, which are widely employed in clinical practice but remain under-investigated locally.

### 5.4 Non-pharmacological interventions

Psychological interventions, including cognitive-behavioral therapy and gut-directed hypnotherapy have shown promise in several studies worldwide. Further studies should explore the effectiveness and cost-efficiency of these therapies in the Vietnamese context as well as integrating psychological support into routine IBS management.

While the low-FODMAP diet has shown promise in Western countries, its application in Vietnam remains unexplored. More studies are needed to assess the feasibility and effectiveness of low-FODMAP diets in Vietnamese population, as well as their impact on IBS symptoms.

### 5.5 Economic burden

Despite the rising prevalence of IBS in Vietnam, the economic burden of IBS remains a largely unexplored area of research in the current literature. To date, there is a paucity of studies quantifying the direct and indirect costs associated with IBS management. Therefore, future research should prioritize cost-of-illness analyses to estimate the overall economic burden of IBS, particularly regional disparities in healthcare access and expenditure. In addition, cost-effectiveness analysis of current IBS management strategies should also be undertaken to inform resource allocation and optimize clinical decision-making within the Vietnamese healthcare system.

## 6 Study limitations

First, although a comprehensive search strategy was applied across multiple databases, relevant studies published in non-indexed local journals or in institutional repositories may have been missed.

Second, this review did not perform a formal critical appraisal of the methodological quality of included studies, consistent with the general purpose of scoping reviews. As such, findings reported from included studies should be interpreted with caution, particularly those derived from small sample sizes, single-center studies, or those lacking control groups and randomization.

Lastly, this review focused on studies published between September 2020 and July 2025 to build upon the previous review by Quach et al. As a result, studies published shortly before or after this timeframe may have been excluded, especially those pending publication or not yet indexed.

## 7 Conclusion

In summary, irritable bowel syndrome (IBS) is a rising health concern in Vietnam, with varying prevalence across different populations. The condition is associated with poor quality of life and comorbidities such as anxiety and depressions. Other overlapping conditions documented includes *H. pylori* infection, SIBO, and colonic abnormalities. Current treatment approaches primarily involve pharmacological agents.

Although progress has been made in research on IBS in Vietnam in recent years, several key gaps remain. These include the need for consistent diagnostic criteria, stronger clinical evidence on treatment effectiveness and safety, especially psychological and dietary interventions, and studies that explore the economic impact of the disease. These findings have important implications not only for clinical practice but also for health policy and resource allocation in low- and middle-income countries.

## Data Availability

The original contributions presented in the study are included in the article/supplementary material, further inquiries can be directed to the corresponding author.
